# Identification of Potential Sources of Mercury (Hg) in Farmland Soil Using a Decision Tree Method in China

**DOI:** 10.3390/ijerph13111111

**Published:** 2016-11-09

**Authors:** Taiyang Zhong, Dongmei Chen, Xiuying Zhang

**Affiliations:** 1 School of Geographic and Oceanographic Sciences, Nanjing University, 163 Xianlin Road, Nanjing 210023, China; taiyangzhong@163.com; 2 Department of Geography and Planning, Queen’s University, Kingston, ON K7L 3N6, Canada; chendm@queensu.edu; 3 Jiangsu Provincial Key Laboratory of Geographic Information Science and Technology, International Institute for Earth System Science, Nanjing University, Nanjing 210093, China; 4 Jiangsu Center for Collaborative Innovation in Geographical Information Resource Development and Application, Nanjing 210023, China

**Keywords:** sources of Hg concentrations, farmland soil, decision tree, China

## Abstract

Identification of the sources of soil mercury (Hg) on the provincial scale is helpful for enacting effective policies to prevent further contamination and take reclamation measurements. The natural and anthropogenic sources and their contributions of Hg in Chinese farmland soil were identified based on a decision tree method. The results showed that the concentrations of Hg in parent materials were most strongly associated with the general spatial distribution pattern of Hg concentration on a provincial scale. The decision tree analysis gained an 89.70% total accuracy in simulating the influence of human activities on the additions of Hg in farmland soil. Human activities—for example, the production of coke, application of fertilizers, discharge of wastewater, discharge of solid waste, and the production of non-ferrous metals—were the main external sources of a large amount of Hg in the farmland soil.

## 1. Introduction

Mercury (Hg) is considered a global pollutant, and the effects of Hg on ecosystems and human health are well documented [1,2,3]. The concentrations of Hg in farmland soil are greatly influenced by parent material and soil properties, including organic matter, soil microbes, and soil pH [4,5,6], as well as human activities, such as non-ferrous mining, petroleum refining and fossil fuel combustion, discharge of wastes from industry production, and applications of fertilizers [7,8,9]. Some studies suggested that anthropogenic sources are leading to a general increase in Hg on local, regional, and global scales [10,11,12]. 

Efforts to identify the sources of Hg in farmland soil are of great significance for contamination prevention and control in the soil-crop system [13]. To search for possible Hg sources of heavy metal concentrations in soil, a number of methods have been proposed, including statistical and geochemical, regulatory reference value, and GIS-based methods [14,15,16,17,18]. Since soil Hg concentration is the result of multiple source interactions, the analysis should consider all of these sources and their interactions. The present study aims to develop a method to estimate the potential sources for Hg in farmland soil, based on a decision tree analysis (DTA). Compared to other statistical tools, DTA offers the following advantages: (a) it is able to handle different types of variables including numeric, categorical, ratings, and survival data; (b) it is able to identify nonlinear relationship and high-order interactions; and (c) results from DTA can be interpreted relatively easily [19,20]. 

However, there have only been a few studies using DTA in assessing soil pollution by heavy metals [19,21,22,23]. A classification and regression tree (CART) method was used to simulate soil Cu concentration and concluded that low soil Cu accumulation was driven by terrain characteristic, agriculture land uses, and soil properties, while high Cu resulted from industrial and agricultural activities [21]. CART was used to investigate the sorption and retention of heavy metals by soil [22]. Hu and Cheng used a conditional inference tree (CIT) to study the sources of heavy metals and concluded that Cd, Zn, Cu, Pb, and Cr in surface soils were largely by anthropogenic sources, whereas As, Ni, and Hg mostly originated from the soil parent materials [19]. In the above studies, the DTA methods were used under the cases in which the data pairs were at point scale, where the soil Hg addition was mainly from a single source. 

On a provincial scale, the sources of soil Hg accumulation from high Hg concentrations in parent materials and human activities are interactive and complicated. Moreover, the identification of Hg sources on the provincial scale is helpful to provide a scientific basis for the government to enact effective policy to prevent further Hg pollution and carry out remediation methods. This study aims to: (a) identify the natural and anthropogenic sources of Hg accumulations in farmland soils on the provincial scale in China; and (b) evaluate the influencing magnitudes of these factors on Hg present in the soil, based on a decision tree method. 

## 2. Data Collection and Methods

### 2.1. Hg Concentrations in Farmland Soil in China

The data on Hg concentration in farmland topsoil (0–20 or 0–15 cm) were collected from the studies published during 2005–2013 throughout China. The process of selecting relevant papers and the data records have been described in the work of Zhang et al. [24,25]. In total, 388 peer-reviewed articles on Hg concentrations are collected. The selected Hg concentrations in farmland soil are then grouped on the provincial scale as an area-weighted mean, based on the following equation:

(1) $$C_{m}=\frac{C_{i}\times A_{i}}{\sum_{i=1}^{n}A_{i}}$$

where *C_m_* is Hg concentration in farmland soil in province *m*, *n* is the number of the published papers in province *m*, and *A_i_* is the investigated area in the *ith* data record.

### 2.2. Backgrounds of Soil Hg Concentrations and Statistic Data on Provincial Scale

The magnitude of Hg background depends on the composition of the parent rock material from which the soil was derived. Mercury is an extremely rare element in Earth’s crust, having an average crustal abundance by mass of only 0.08 parts per million [26]. Hg is found either as a native metal (rare) or in cinnabar, living stonite and other minerals, with cinnabar (HgS) being the most common ore [27]. The Hg backgrounds on the provincial scale were collected from the book of Soil Backgrounds in China [28], which collected soils from A, B, and C horizons of 4095 soil profiles and analyzed for concentrations of 13 elements involved of Hg [29]. These soil samples were developed from 19 kinds of parent materials in China, on which the Hg concentration ranged from 0.03 mg/kg on sedimentary red sandstone to 0.177 mg/kg on marine sedimentary parent material [28]. 

The reasons for selecting human actives on influencing Hg concentrations in soil are followed the previous studies and whether these data are accessible. The continuous application of wastewater and solid wastes into the farmland would lead to the high Hg concentrations in soil [30,31]. The long-term application of excessive fertilizers, organic manures, and pesticides to the farmland have been approved to introduce additional Hg concentration into soils [9,32,33]. The production procedures of coke, paper, steel, and glass could emit Hg into the environment, and eventually lead to high concentrations of Hg in soils [7,34,35]. Particularly, the mining and smelting activities on non-ferrous metals could emit a large amount of Hg into the atmosphere, water, and soil [36,37], and Hg could be accumulated in the soil through atmospheric depositions and wastewater irrigations. The anthropogenic attributes are from the Statistic Book of China [38]; including discharge of wastewater per farmland land (DWW); discharge of solid waste per farmland land (DSW); pesticide application rate (PAR); fertilizer application rate (FAR); irrigation rate (RR); production of coke (PFC); the total production of paper, steel, and glass (PI); non-ferrous metal reserves (NFMR); and combustion of fossil fuel (CFF). 

### 2.3. Decision Tree Method to Drive Potential Hg Sources in Soil on Provincial Scale

The decision tree method is used to identify the potential sources of Hg present in the soil. Understanding the impact of soil and landscape properties and human activities on Hg content could be used to estimate the Hg addition in the soil. The detail information on the decision tree model, C5, is described in the papers of [39,40]. 

The input to the model of C5.0 is a training set of records, each of which is a set of attribute values tagged with a decision label. In this study, the data pairs of Hg addition and the potential sources of soil Hg were used as inputs. Hg accumulation was calculated as follows:

*AC_m_* = *C_m_* − *B_m_* (2)

where *AC_m_* is the Hg addition in province *m*, *C_m_*, and *B_m_* are the Hg concentration and background value in province *m* [28,41,42]. The unit for *AC_m_*, *C_m_*, and *B_m_* is mg/kg.

To construct the decision tree to simulate the human activities on Hg concentration in soil, nine attributes are selected. The C5.0 can pre-selected a subset of the attributes that will be used to construct the decision tree by the function of “winnowing”. The remaining attributes are then listed in order of importance, where the numerical importance shown for each attribute is the estimated percentage increase in error rate or misclassification cost that would result from removal of that attribute. 

## 3. Results and Discussion

### 3.1. Statistic Information of Hg Concentration in Farmland Soil

The statistic information of Hg concentration in farmland soil on the provincial scale is described in Table 1. The investigated areas were spatially distributed in 30 provinces, municipalities, or districts, covering all of the mainland area of China except the Tibet Autonomous Region and Taiwan Province. The number of studies within a province ranged from 2 to 27, and the number of investigated samples ranged from 31 to 31,211.

The range of area-weighted Hg concentration in farmland soil was from 0.017 mg/kg to 0.554 mg/kg. Some of the provinces had higher Hg concentrations than the reference II of 0.300 mg/kg under soil pH < 7.5, indicating that these areas faced high Hg pollution risk. The high Hg concentrations occurred in Tianjin, Xinjiang, Hunan, Guangxi, Guizhou, and Fujian, with the value higher than 0.200 mg/kg. The Hg concentration in the farmland soil of Guangdong, Hubei, Jiangxi, Liaoning, Shanghai, Sichuan, Yunnan, and Zhejiang ranged from 0.100 mg/kg to 0.200 mg/kg. The remaining provinces had Hg concentrations under the value of 0.100 mg/kg. 

### 3.2. The Influence of Initial Hg Concentration in Parent Materials on Hg Concentration in Farmland Soil

**Figure 1:**
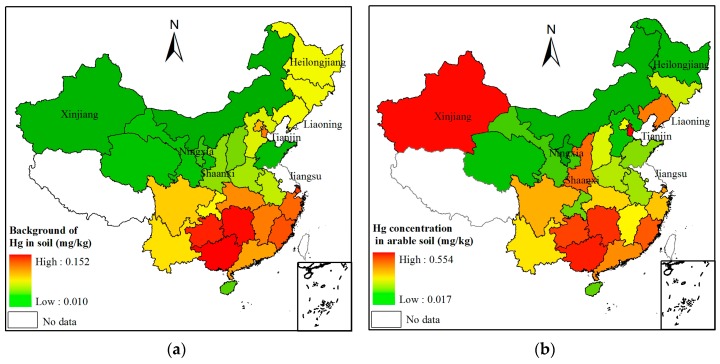
Spatial distribution of (**a**) background of Hg concentration in soil [28,41,42]; and (**b**) area-weighted average of Hg concentration in arable soils in the mainland of China. Note: in this section, Jiangsu Province was not involved since the background value of Hg is 0.287 mg/kg, which much different from those in other provinces. The area-weighted Hg concentration in the arable soil in Jiangsu Province is 0.1148 mg/kg.

To illustrate the contribution of physical initial concentration in parent materials on current Hg concentrations in farmland soil, the spatial distribution of background of soil Hg, and the area-weighted mean of Hg in farmland soil are illustrated in Figure 1. The background concentration of Hg in soil showed an obvious spatial trend of soil Hg concentrations decreasing from the south to the north. The area-weighted average of Hg concentration in farmland soil had a similar spatial trend, but Hg concentrations in some provinces disrupted such spatial variations.

**Figure 2:**
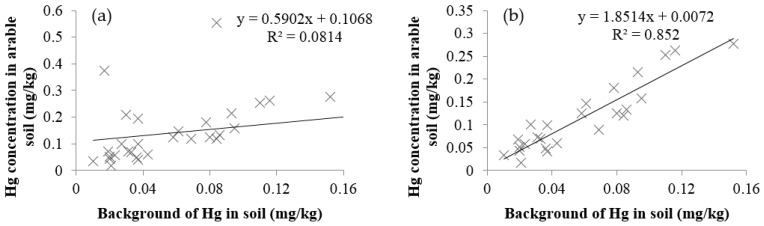
Scatter plots of background of Hg in soil and Hg concentration in arable soil (**a**) the whole data set; and (**b**) after removing four data pairs.

The relationship between the background concentrations of Hg in soil and the area-weighted Hg concentration in farmland soil on a provincial scale were examined (Figure 2). From Figure 2a, it was evident that several data deviated from the primary trend, indicating the Hg concentrations in these provinces were greatly influenced by external sources. If these provinces were removed from the data set (Figure 2b), the background concentrations of Hg dominated in farmland soils of the remaining provinces in farmland soil in the remaining provinces. 

The excluded four provinces were Liaoning, Shaanxi, Tianjin, and Xinjiang, which had relatively low backgrounds yet high Hg contents in farmland soil. Liaoning Province had a lot of non-ferrous mining and smelting activities, high farmland irrigation rates with wastewater, and well developed heavy industries [30,43,44]. In Xinjiang and Tianjin, the high Hg contents in farmland soil were mainly introduced by the sewage irrigation [45,46,47]. The high Hg concentration in Shaanxi Province might be due to non-ferrous mining and smelting activities and sewage irrigation [37,48]. 

### 3.3. The Influence of Human Activities on Hg Accumulation in Farmland Soil

#### 3.3.1. Description of the Decision Tree 

To assess the importance of potential human sources on Hg accumulation in farmland soil on a provincial scale, the decision tree method of C5.0 was used. From Figure 2, Hg concentrations in most provinces were higher than the according backgrounds except for in Ningxia (−0.0035 mg/kg) and Heilongjiang (−0.0016 mg/kg), indicating these areas were influenced by exterior factors. Hg concentrations were seldom influenced by the human activities in these two provinces since the averaged concentrations of Hg from 2006–2013 were even lower than their corresponding background values. Certainly, the limited collected samples in these two provinces might introduce some uncertainty on the Hg concentration, and led to the negative values. The accumulations of Hg in the remaining provinces ranged from 0.0040 mg/kg (in Hebei Province) to 0.4702 mg/kg (in Tianjin). The higher Hg accumulations represented stronger influences from external sources. 

**Figure 3:**
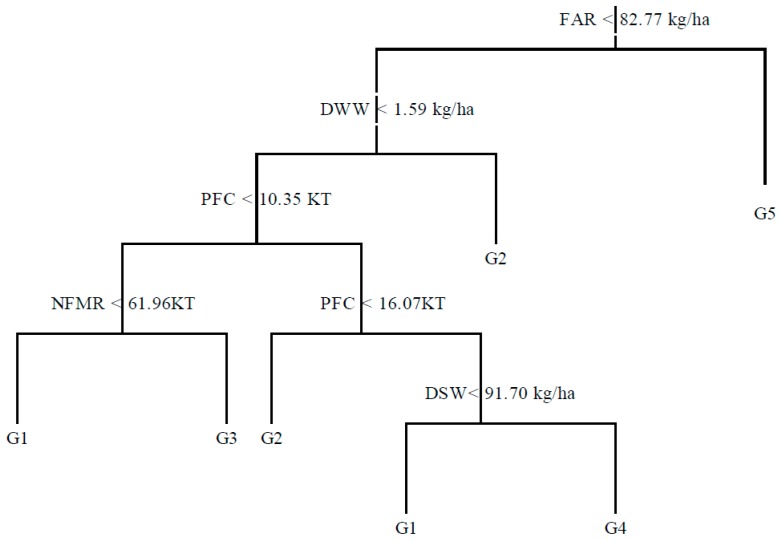
Decision tree on Hg concentration in farmland soil in China.

According to the ranges of Hg accumulations (*AC_m_*) in farmland soil, the 29 provincial cases were grouped into five grades (G1–G5). Hg accumulation in the five grade ranges were 0.004–0.050 mg/kg, 0.051–0.100 mg/kg, 0.101–0.150 mg/kg, 0.151–0.2000 mg/kg, and 0.201–0.470 mg/kg. The simulated tree included eight nodes (Figure 3). In the decision tree, 2, 2, 1, 1, 1 branches were for the grades from G1 to G5. The accuracy of the C5.0 training process correctly matching to their respective classes was 89.70%. Among the 13 samples in G1, 12 provinces were correctly simulated for G1, and 1 for G3. Among the six provinces in G2, five were correctly simulated into G2, while one was for G4. Among the six provinces in G3, five were correctly classified in G3, and one for G2. The four provinces in G4 and G5 were correctly simulated into their according grades.

#### 3.3.2. Evaluation of the Relative Importance of Factors on Hg Accumulation 

The method C5.0 provides the relative importance of independent variables on the soil Hg concentration. The decision tree finally selected five attributes of fertilizer application rate (FAR), discharge of wastewater (DWW), production of coke (PFC), non-ferrous metal reserves (NFMR), and discharge of solid waste (DSW), from the nine initially selected attributes. This selection does not mean that the unselected parameters had little contribution to the Hg accumulation in arable soil. The reason for these parameters not being selected in the decision tree might be that they had a non-essential effect on Hg accumulation. 

The misclassification error showed that PFC was most important to classify soil Hg accumulation (omitting PFC increased misclassification error to 24.1%), followed by the parameters of FAR, DSW, and DWW (misclassification error to 17.2%), and NFMR (misclassification error increased to 13.8%). The high Hg concentration in the arable soil in the mining area of non-ferrous metals or fossil fuels is mainly due to mineral excavation, ore transportation, smelting, and refining in these areas, as well as disposal of the tailings and wastewater around mines [49,50,51]. Although the activities of non-ferrous metal mining—such as copper, lead, and zinc—could introduce larger amounts of Hg into the environment than the fossil mining [37,51,52], the contribution of fossil mining was higher than non-ferrous metals on the accumulation of Hg in farmland soil because the production of fossils were much higher than the non-ferrous metals. For example, coal production was 36.8 × 10^8^ T, and the total production of non-ferrous metals was 4.05 × 10^7^ T in 2013 in China [53].

The effect of fertilizer applications showed high contribution on Hg accumulations in farmland soil. Hg concentrations detected in fertilizers commonly used in agricultural activities ranged from 0 to 5.1 mg/kg [33]. Particularly, the content of Hg in calcium superphosphate was 5–10 times higher than the limit of grade II soil in Environmental Quality Standard for soils in China (GB 15618-1995). The other study showed that the phosphorous fertilizers could influence soil Hg concentrations to some extent where has a low Hg background value in soil [9]. Thus the application of liquid and soil manure or inorganic fertilizers could introduce a large amount of Hg into farmland soil since fertilizer application is a common agricultural practice [32].

The discharge of solid wastes or wastewater had great effect on the Hg accumulation in farmland soil. The wastes from the pharmaceutical, paper, electric, and chemical industry plants often contained a large amount of Hg [54]. In China, most of the irrigated water was untreated sewage or effluents of primary treatments, and the municipal wastewater and industrial wastewater were not separated in many cases. Thus, Hg could enter into the soil through direct sewage irrigation or atmospheric diffusion or surface runoff flushing or weathering from the solid wastes [55]. In China, irrigation with sewage was becoming a common practice due to shortage of fresh water. This situation was especially common in the urban and suburban areas [47,56] and the arid or semiarid areas [45]. The total area of sewage area in China was about 40,000 km^2^. 

#### 3.3.3. Decision Rules for Hg Accumulation in Farmland Soil

FAR had the determinant effect on whether Hg accumulation reached a level of G5.0. When FAR was higher than 82.77 kg/ha, the soil would accumulate to a great amount of Hg in farmland soil. In fact, the two regions also had high irrigation rates (higher than 75%) [38], and the sewage irrigation might introduce a considerable amount of Hg into farmland soil. 

When FAR was lower than 82.77 kg/ha, and DWW was higher than 1597 T, such as the megacity of Beijing and Shanghai, Hg would be introduced into farmland soil since sewage might be used for irrigating farmland soil. As a result, Hg pollution problems were broadly noticed in soil irrigated with the reclaimed water. Under the conditions of FAR lower than 82.77 kg/ha, DWW lower than 1597 T, PFF lower than 1035 KT, and NFMR lower than 6196 KT, Hg accumulations would be in G1. While under the same condition of FAR, DWW, EP, but with NFME greater than 6196 KT, Hg accumulations would reach G3. This also indicates that mining and smelting activities introduced large Hg concentrations into farmland soils. 

When FAR was lower than 82.77 kg/ha, DWW was less than 1.57 KT, and the EP was between 1036 KT and 1607 KT, Hg accumulation would be in G2; while under the same condition of FAR, DWW, EP, if the DSW were lower than 91.70 kg/ha, Hg accumulation would be in G1; otherwise Hg would be in G4. The solid wastes from industry development, such as battery production, contained high Hg contents. If these wastes were not properly disposed, they would pollute the peripheral farmland soils. 

### 3.4. Limitations and Uncertainties

The method of decision tree C5.0 identified the sources of soil Hg concentrations on the provincial scale and found the complicated relationship between the Hg concentrations and the sources from parent materials and human activates. However, there were some limitations when using C5.0 to simulate the sources of Hg concentration in soil. First, the simulation results showed instability [57]. Even a small change in the input data would cause large variations in the simulated tree. Second, C5.0 has the inadequacy in applying regression for predicting continuous values. Although the decision tree method has been used to assess soil Cu content (divided into six grades) considering the human activities and gained a better estimation result than Kriging, it still can only simulate the scalar data [21]. 

## 4. Conclusions

This study identified the potential sources of Hg in the farmland soil on a provincial scale in China based on the soil Hg concentrations from published papers, the background Hg concentrations from parent materials, and the statistical data relevant to Hg sources. The decision tree gained a reliable result on simulating the interactive effects of the multiple sources of Hg in soil. The natural factors showed a strong influence on Hg concentration in farmland soil on the province scale, while the human activities changed such spatial trends. The human activities of production of coke, application of fertilizers, discharge of wastewater, discharge of solid waste, and production of non-ferrous metals, led to the high accumulation of Hg in farmland soil in China. 

## Figures and Tables

**Table 1:** Statistic information of Hg concentration in farmland soil on provincial scale.

Provinces, Municipalities, and Districts	Number of Studies	Investigated Area (km^2^)	Number of Soil Samples	Area-Weighted Hg Concentration in Farmland Topsoil (15 or 20 cm)
Anhui	16	10,329	9780	0.081
Beijing	10	8374	1378	0.118
Fujian	10	17,643	1508	0.214
Gansu	20	6771	691	0.053
Guangdong	27	23,885	2076	0.180
Guangxi	9	14,752	662	0.277
Guizhou	24	4004	3449	0.252
Henan	7	14,882	747	0.058
Hebei	12	3068	1552	0.073
Heilongjiang	11	141,087	25,519	0.035
Hubei	13	2839	1340	0.125
Hunan	19	3218	1211	0.271
Jilin	7	14,500	2188	0.099
Jiangsu	27	84,871	31,211	0.099
Jiangxi	7	14,281	10,611	0.120
Liaoning	21	8242	7989	0.117
Neimeng	6	10,687	2012	0.034
Ningxia	2	328	31	0.017
Qinghai	2	282	207	0.043
Shandong	23	46,479	16,075	0.071
Shanxi	16	2624	2643	0.100
Shaanxi	17	3470	1385	0.209
Shanghai	6	451	933	0.157
Sichuan	19	6631	2063	0.152
Tianjin	7	449	295	0.554
Xinjiang	11	1242	666	0.375
Yunnan	12	1311	608	0.124
Zhejiang	21	26,544	5326	0.133
Chongqing	6	2640	1138	0.060
